# Sustainable diets and functional foods for the prevention of cardio-metabolic diseases and sustainable development goals of the UNO. An international consensus of scientific statement of the international college of nutrition and 28th world congress on clinical nutrition, Bogor, Indonesia

**DOI:** 10.1186/s12872-026-05557-7

**Published:** 2026-07-07

**Authors:** Hardinsyah Ridwan, Sulaeman Ahmed, Ram B. Singh, Saibal Chakravorty, Oleg Medvedev, Rana G. Singh, Harpal S. Buttar, Ajay Agarwal, Lekh Juneja, Douglas W. Wilson, Delphi Panel: International College of Nutrition, Amitabh Yaduvanshi, Kuldeep Dhar, Sanjay Mahajan, Anuj Maheshwari, Narsingh Verma, Meenakshi Jain, Fabien De Meester, Jitendra Singh, Branislav Milovanovic, A. K. Shukla, Dominik Pella, Najah Hadi, Shashank Joshi, Agnieszka Wilczynska, Ghazi Halabi, Galal Elkilany, Mahmood Moshiri, Germaine Cornelissen, Jarmila Siegelova, Jaipaul Singh, Osama Elmaraghi, Fabiola Sozzi, Narsingh Verma, Ernest Adeghate, Sibel C. Enar, Kairola Rakhimov, Abuova Zhanar, Adrian Isaza, Arsha Moshiri, Sergey Chibisov, Ghizal Fatima, Duo Li, Valeria Pergola, Krasimira Hristova, Jan Fedacko, Banshi Saboo, R. K. Goyal, Zuzana Sumbalova, Anna Gvodjakova, Jana Muchová, Shallendra Vajpayee, MA Manal, Rajeev Gupta, Preksha Chaudhury, Meenakshi Singh, Rukam Singh, Ekasit Onsaard, Wiriya Phomkong, Sundeep Mishra, Aminad Megomedova, Megomed Magomedov, A. K. Chauhan, J. P. Sharma, Sarthak Sharma, Istvan Telessy, Fidela Nailan FP, Alvira Sulastiyo, Ali Magomedov, Beatrice K. Lisdwiyani

**Affiliations:** 1 Department of Food and Nutrition, IPB University, Bogor, Indonesia; 2 Department of Community Nutrition, Faculty of Human Ecology, IPB University, Bogor, Indonesia; 3 Halberg Hospital and Research Institute, Moradabad, India; 4 Metro-Multispeciality Hospital, Noida, India; 5 Faculty of Basic Medicine, Lomonosov Moscow State University, Moscow, Russia; 6 Heritage Institute of Medical Sciences, Varanasi, India; 7 Department of Pathology & Laboratory Medicine, Faculty of Medicine, University of Ottawa, Ottawa, Canada; 8 Department of Internal Medicine, Fortis Hospital, Noida, India; 9 Kameda Seiko Company Limited, Tokyo, Japan; 10 School of Medicine, Pharmacy and Health, Durham University, Formerly, UK; 11 Head of Cardiology, Holy Family Hospital, New Delhi, India; 12 Kailash Hospital & Heart Institute, Noida, India; 13 Department of Medicine, Hind Institute of Medical Sciences, Ataria, Sitapur India; 14 Max Super Speciality Hospital, New Delhi, India; 15 The Tsim Tsoum Institute, Krakow, Poland; 16 Department of Medicine, King George’s Medical University, Lucknow, India; 17 University Clinical Center, Bezanijska Kosa, University of Belgrade, Belgrade, Serbia; 18 Department of Medicine, Kailash Hospital and Research Center, Noida, India; 19 University Research Park, PJ Safaric University, Kosice, Slovakia; 20 University of Kufa, Najaf, Iraq; 21 Department of Endocrinology, Leelawati Hospital, Mumbai, India; 22 The Tsim Tsoum Institute & Wyzsza Szkoła Ksztalcenia Zawodowego, Wroclaw, Poland; 23 Halberg Cardiac Center, Alay, Lebanon; 24 Sentara Northern Virginia Medical Center, Woodbridge, VA USA; 25 International College of Cardiology, Richmond Hills, Canada; 26 Halberg Chronobiology Center, University of Minnesota Medical School, Minneapolis, MN USA; 27 Dept. Sports Medicine and Rehabilitation, Masaryk University, Brno, Czech Republic; 28 School of Pharmacy and Biomedical Sciences, University of Central Lancashire, Preston, England UK; 29 Diabetic Center, Jaahra, Kuwait; 30 Department of Cardiology, Milan University, Milano Majoree Polyclinico, Milan, Italy; 31 Hind Institute of Medical Sciences, Ataria, Sitapur India; 32 Department of Human Anatomy, College of Medicine and Health Sciences, United Arab Emirates University, Al Ain, UAE; 33 Altinbas University School of Medicine, Istanbul, Turkey; 34 Kazakh National Medical University, Almaty, Kazakhstan; 35 Everglade University, Tampa, FL USA; 36 RUDN University, Moscow, Russia; 37 Department of Biotechnology, Era University, Lucknow, India; 38 Institute of Nutrition and Health, Qingdao University, Qingdao, China; 39 Department of Cardiology, University of Padua, Padua, Italy; 40 Center for Cardiovascular Disease, Sofia, Bulgaria; 41 Saboo Diabetes Research Center, Ahmedabad, India; 42 Formerly, Delhi Pharmaceutical Sciences, & University, New Delhi, India; 43 Faculty of Medicine, Institute of Medical Chemistry, Biochemistry and Clinical Biochemistry, Comenius University in Bratislava, Bratislava, Slovakia; 44 Government Medical College, Surat, India; 45 School of Pharmacy, University of Central Lancashire, Preston, UK; 46 Spectrum Medical Center, Abu Dhabi, UAE; 47 Max Super Specialty Hospital, Noida, India; 48 CSIR, New Delhi, India; 49 Junagarh Agri University, Junagarh, India; 50 Faculty of Agriculture, Ubon Ratchathani University, Ubon Ratchathani, 34190 Thailand; 51 Indigenous Food Research and Industrial Development Center, Ubon Ratchathani University, Ubon Ratchathani, 34190 Thailand; 52 Uzala Cignus Bright Star Hospital, Moradabad, India; 53 Lomonosov Moscow State University, Moscow, Russia; 54 Department of Food and Nutrition, Institute of Agriculture, BHU, Varanasi, India; 55 Department of Pharmaceutics, Faculty of Pharmacy, University of Pécs, Pécs, Hungary; 56 Department of Nutrition, Fidela Nailan Faza Prasetyazi, IPB University, Bogor, Indonesia; 57 Department of Nutrition, Alvira Sulastiyo, IPB University, Bogor, Indonesia; 58 Institute of Engineering, Ali Magomedov, Makhaskala, Russia; 59 Faculty of Medicine, Beatrice Kunthi Lisdwiyani, Universitas Airlangga, Surabaya, Indonesia

**Keywords:** Diet, Nutrition, Cardiovascular diseases, Type 2 diabetes, Stroke, Cancer, Preconception, Earth, Environmental degradation

## Abstract

**Background:**

The United Nations Organization (UNO) has proposed achieving specific Sustainable Development Goals (SDGs), including poverty, hunger, health, education, equality, climate change, and the need for sustainable development, while protecting the planet Earth from environmental degradation. The expert group of the International College of Nutrition aims to emphasize the merits of sustainable diets and traditional plant based foods for the primordial prevention of cardio-metabolic Diseases (CMDs) and other chronic diseases in this review, to achieve these goals.

**Methods:**

A narrative review was conducted to identify articles related to cardiovascular diseases (CVDs), obesity, diabetes, and cancer, using databases from the World Health Organization (WHO), Google Scholar, MEDLINE (PubMed), Web of Science, and EBSCO, along with additional secondary sources and a search of grey literature. Opinions of experts were also sought, and views of all authors were obtained as outlined in this document.

**Results:**

It seems that education, in particular health education and motivation, apart from cultural factors, are crucial for achieving total health, and the SDGs of the UNO. The prevalence of unhealthy behaviours and risk factors for most of the CMDs and other chronic diseases is rapidly increasing in low- and middle-income countries, due to the ongoing economic development, leading to rapid changes in diet and lifestyle. There is some decline in cardiovascular disease (CVD) mortality in high-income countries due to education and learning of preventive strategies, resulting in a reduction in mortality due to these diseases. However, CMDs and cancer remain the significant causes of morbidity and mortality. During and after World War II, food scarcity persisted from 1940 to 1965 in most countries, accompanied by a low risk of cardiovascular disease (CVD) and diabetes. The rapid increase in industrialization and urbanization has been linked to environmental degradation on planet Earth, a decline in fertile, healthy soil, and sustainable, functional farming practices, resulting in a decrease in the production of nutritious foods. These alterations in food availability are associated with an increased production of unhealthy, energy rich foods and Western-type dietary patterns, which in turn increased the risk of non-communicable diseases (NCDs). The health of the planet and its people is at risk. There is a deterioration in the global standard of natural systems that support life on Earth, which is exacerbating energy, food, and water insecurity, and increasing the risk of disease, disaster, displacement, and conflict. Recent advice about the safe corridor on the planet can enhance research on planetary boundaries and Earth-system aspects of the SDGs. It appears that poverty, lack of education, and inadequate health education and motivation, possibly due to ineffective policies by national and local governments, are significant determinants of the increased risk of these diseases. In urban areas of lower and middle-income countries, as well as in immigrant populations in high-income countries, CMDs and other chronic diseases are significantly higher than they are in some of the high-income populations. Improvements in soil and the promotion of functional farming, along with an emphasis on the food industry, are urgently needed to support plant-based, low-animal food traditional food diets. Traditional foods are feasible and affordable in every country. The use of high-protein substitutes for animal-derived foods, such as soybean products such as tofu and tempe, millets, beans, lentils, peas (400 g/day), green vegetables, fruits, and nuts (400 g/day), as well as vegetable oils, low-fat dairy products, and culinary spices, with low meat (sea-foods) may be beneficial in promoting health and preventing NCDs. Health education and promotion of healthier lifestyles and behaviours during the preconception period, in utero life, and the postnatal development and infancy stages may lead to healthy eating patterns, contributing to primordial prevention of NCDs. A wild-type omega-6/3 diet rich in flavonoids, folate, and other nutrients, along with other interventions may mitigate epigenetic risk variations at the individual level across all subgroups, from the preconception period to the elderly stages.

**Conclusions:**

These findings may necessitate revisions to the existing guidelines proposed for sustainable functional farming and sustainable plant based diets. Eating 400 g/day of vegetable, fruits, and nuts and another 400 g/day of whole grains, with spices (20-50 g/day) and meat as condiment (50 g/day), along with 30-50 g/day of vegetable oils, can prevent CMDs and other chronic diseases, and promote health to serve the SDGs of the UNO. Cohort studies and randomized trial in each country may be necessary to confirm the role of our advice in the prevention of diseases.

## Introduction

The United Nation Organization (UNO) made a universal call in 2015, to establish the Sustainable development goals (SDGs), which are to take action, to protect the planet, to end poverty, and to ensure that people living on earth enjoy peace and prosperity by 2030 [1, 2]. These 17 SDGs that are connected with each other, provide a blueprint for an improved sustainable future for the whole world, which share a text from *Indian Vedas that “people living on Earth is a family”.* The Office of SDGs coordinates the efforts of the FAO, to accelerate the agenda of 2030 [1, 2], through the sustainable transformation of crops to functional farming (growing of crops with nutrient rich foods) and Agri-food systems, by supporting all the countries and stakeholders to implement the SDGs and to increase their capacity [3]. High-quality health system is one of the most important SDG that indicate a time for a revolution using functional foods, for health promotion and disease prevention [4]. *Let thy food thy medicine and thy medicine thy food or the era of food as medicine. (Hippocrates, 5th Century BCE).*

**Fig. 1:**
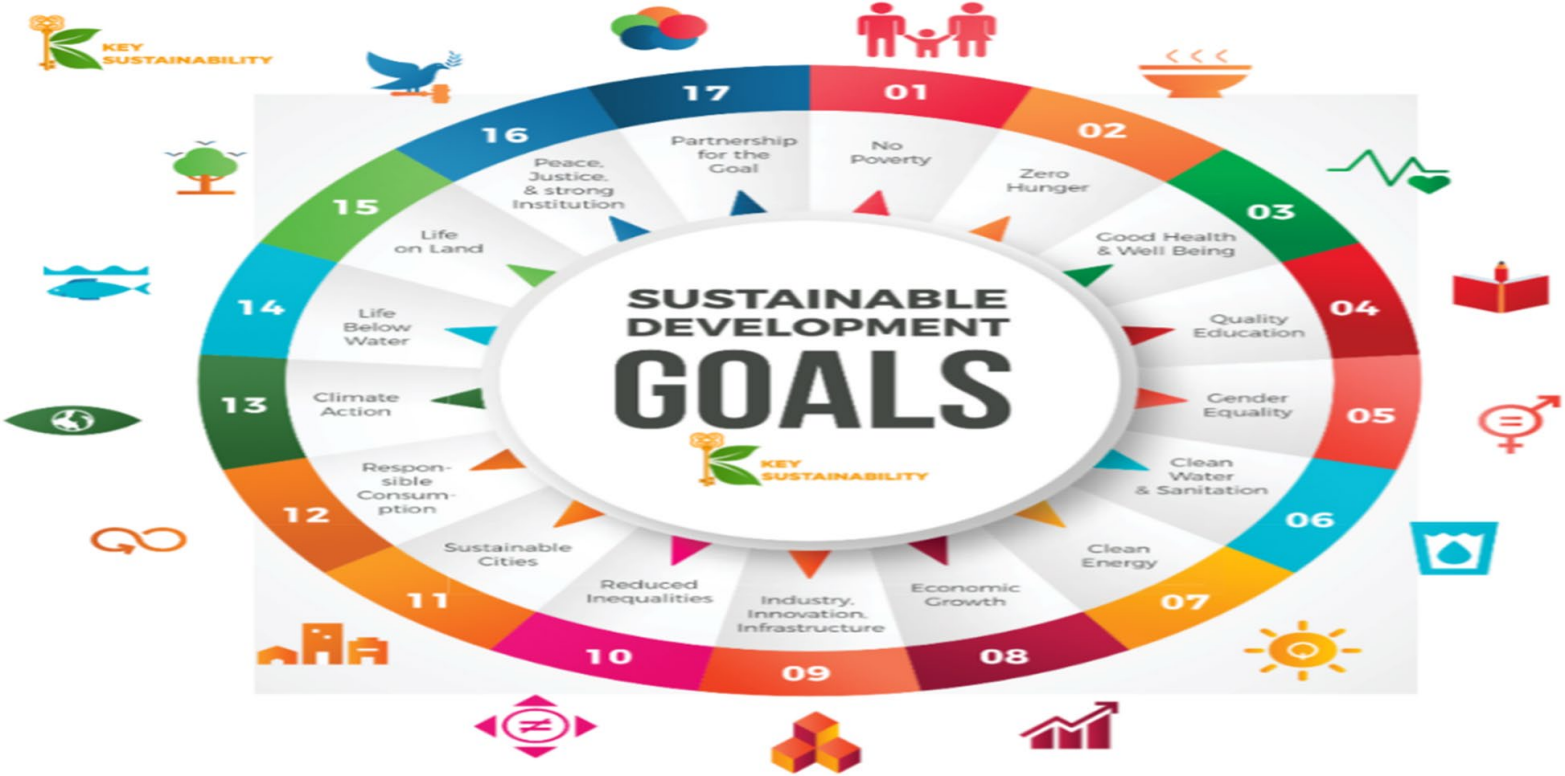
Sustainable development goals of the UNO. (Adapted from reference 1). All the SDGs are shown in various colours in the outer circle

The SDGs address global challenges like health, education, poverty, hunger, equality, climate change, and the need for sustainable development, while protecting the planet earth, which was called mother by the *Babylonians and ancient Indians* and also by modern Indians. The UNO, World Health Organization(WHO) and International College of Nutrition as well as all other health-related organizations have emphasized the value of SDGs. It is important to emphasize on primordial prevention of cardio-metabolic Disease(CMDs) and other chronic diseases for achievement of these goals because all the resources and funds that are generated for human development, are taken away by the drug and health care industry, via treatment of these diseases. Recently, several experts have been exploring the relationship between plant-based diets and the SDGs of the UNO [5–10]. The health of the planet and its people is at risk, due to the deterioration in the global standard of natural systems that support life on Earth. It is exacerbating energy, food, and water insecurity, and increasing the risk of disease, displacement, disaster and conflicts such as terrorism and war [11–13]. The concerned experts emphasize how plant-based diets, can contribute to the achievement of various SDGs by health promotion, with a decline in the impact on the environmental systems, while supporting functional food security [5–7]. This communication aims to highlight SDGs, sustainable diets and traditional food- plant- based affordable diets for health promotion and prevention of NCDs while protecting the earth and environment (Fig. 1).

### The Sustainable Development Goals (SDGs)

The Sustainable Development Goals (SDGs), also known as the Global Goals, are 17 interconnected objectives adopted by the United Nations in 2015 to guide global development efforts through 2030. The Food and Agriculture Organization (FAO) and the World Health Organization (WHO) play key roles in advancing specific SDGs that contribute to a more prosperous and sustainable world. The 2030 Agenda for Sustainable Development, which includes these 17 goals, will shape national development plans over the coming years. These goals address critical global challenges, including poverty, hunger, health, education, equality, climate change, and the need for sustainable development (Table 1).

**Table 1:** The Sustainable Development Goals (SDGs) (Adapted from reference 1)

1. No Poverty: Eradicating poverty in all its forms remains one of humanity’s greatest challenges
2. Zero Hunger: Ending hunger, achieving food security, improving nutrition, and promoting sustainable agriculture
3. Good Health and Well-being: Ensuring healthy lives and promoting well-being at all ages is essential for sustainable development
4. Quality Education: Access to quality education is the foundation of sustainable development
5. Gender Equality: Achieving gender equality is a fundamental human right and a prerequisite for a peaceful, prosperous, and sustainable world
6. Clean Water and Sanitation: Access to clean and safe water is essential for the world we aim to build
7. Affordable and Clean Energy: Energy access is central to addressing nearly every major global challenge
8. Decent Work and Economic Growth: Sustainable economic growth requires creating conditions that enable people to access quality employment
9. Industry, Innovation, and Infrastructure: Investing in infrastructure is vital for sustainable development
10. Reduced Inequalities: Reducing inequalities requires universal policies while focusing on the needs of marginalized populations
11. Sustainable Cities and Communities: Cities are hubs for ideas, commerce, culture, science, and social development
12. Responsible Consumption and Production: Promoting sustainable consumption and production patterns
13. Climate Action: Addressing climate change is a global challenge that affects everyone
14. Life Below Water: Managing ocean resources sustainably is key to a sustainable future
15. Life on Land: Sustainable management of forests, combating desertification, and halting biodiversity loss
16. Peace, Justice, and Strong Institutions: Ensuring access to justice and building accountable institutions at all levels
17. Partnerships for the Goals: Strengthening global partnerships to support sustainable development

### The UN High-Level Meeting on Universal Health Coverage (UHC) 2023

The 2023 UN High-Level Meeting (UN HLM) on Universal Health Coverage (UHC) provided countries and stakeholders with an opportunity to accelerate progress toward achieving health for all [14]. With world leaders having renewed their commitment to achieving UHC by 2030, it is now essential to hold them accountable. All partners in the UHC movement, including governments, parliamentarians, academia, civil society, and the private sector, must ensure that the commitments made at the 2023 UN HLM on UHC translate into concrete actions advancing UHC at the community, regional, national, and global levels. Additionally, India plans to expand health coverage under Ayushman Bharat to all individuals below the poverty line and to all individuals aged 70 years and above. All Member States unanimously recognized that UHC is fundamental to achieving the SDGs, including those related to health and well-being (SDG 3), as well as goals concerning climate change, poverty eradication, access to education, gender equality, and the promotion of peaceful and inclusive societies [14–16].

### United nations organization and world health organization

**Fig. 2:**
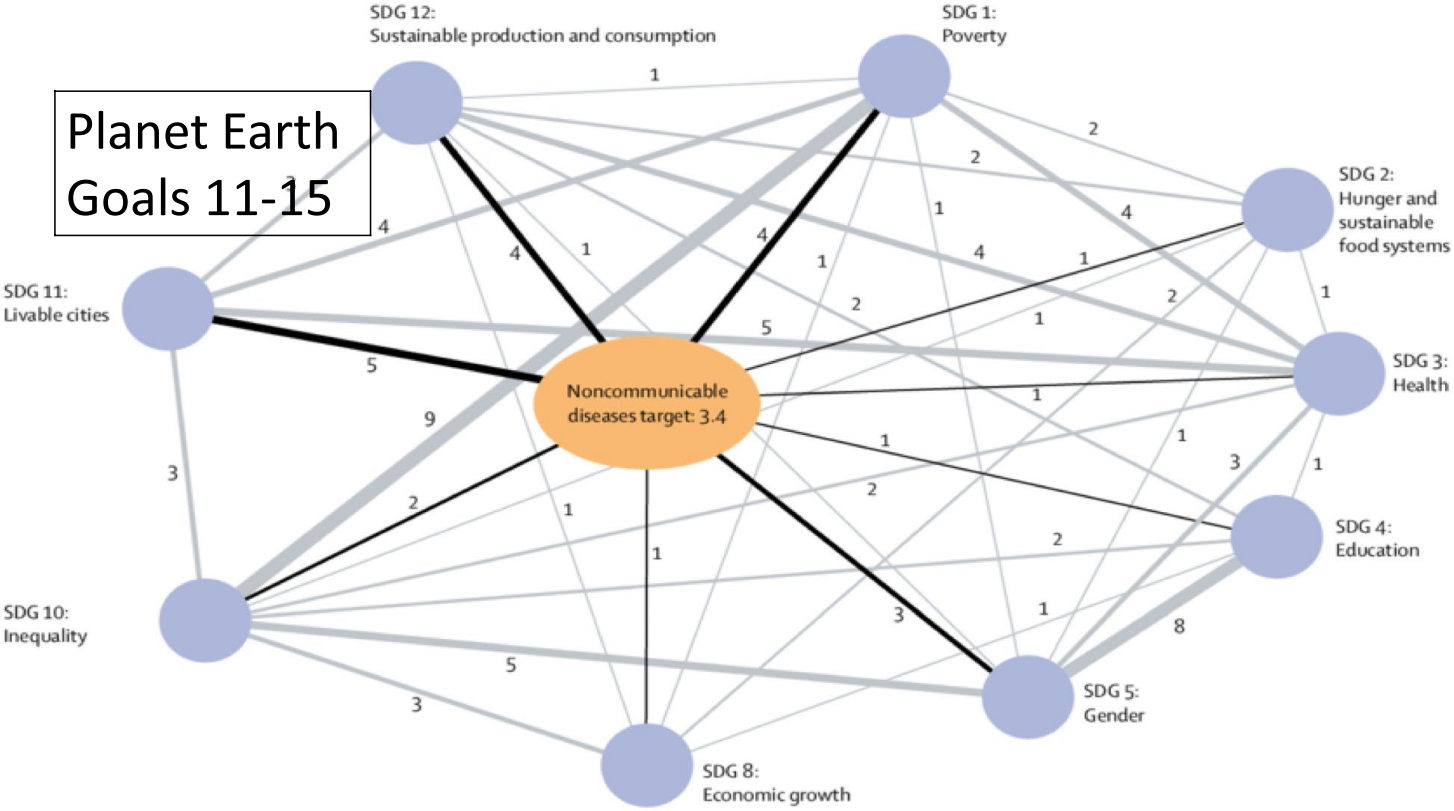
Sustainable development goal 2-4 target non-communicable diseases (NCDs), Goals 2-4 specifically target NCDs and health, Goals 11-15 target planet Earth, while other goals are related to health through their focus on risk factors (Adapted from reference 10) Sustainable development goal 2–4 target non-communicable diseases (Adapted from reference 10)

In September 2000, the United Nations adopted the Millennium Declaration, establishing eight Millennium Development Goals (MDGs) aimed at reducing global poverty by 2015. Of these eight goals, three directly addressed health, focusing on human development without specifying diseases [17]. The worldwide rise of non-communicable diseases (NCDs), including cardiovascular diseases (CVDs) and diabetes, has provided an opportunity to implement actions to curb these epidemics, despite the significant economic burden they impose [17–20]. While there was a declining trend in CVDs in Western countries after 1968, the continued increase in obesity has led to a rise in metabolic syndrome across both developed and developing economies, contributing to the growing burden and mortality associated with NCDs [17]. The International College of Cardiology, Columbus Paradigm Institute, and the International College of Nutrition as well as Eat Lancet Commission, have advocated for the global adoption of wild foods in the diet to protect against cardiovascular diseases and reduce all-cause mortality [18, 19]. It remains necessary to reiterate such recommendations to encourage the food industry and governments to increase the availability of affordable, healthy foods recommended by Eat Lancet [18]. The authors commend the planners of the UN High-Level Meeting on NCDs held in September 2011 for addressing the rising burden of death and disability attributable to CVDs and other chronic diseases in middle- and high-income countries due to rapid dietary and lifestyle changes [19]. Millions of deaths each year result from insufficient health education and inadequate health policies, with CVDs and type 2 diabetes mellitus (T2DM) posing significant challenges to healthcare systems and governments, while also contributing to poverty and hindering human, social, and economic development [9]. Of the 17 SDGs, Goals 2–4 specifically target NCDs and health, Goals 11-15 target planet Earth, while other goals are related to health through their focus on risk factors (Fig. 2).

The United Nations Organization (UNO) and the World Health Organization (WHO) are actively engaged in advancing the SDGs. Adopted by all UN Member States in 2015, the SDGs consist of 17 interconnected goals, with the WHO focusing primarily on SDG 3, which aims to ensure healthy lives and promote well-being for all at all ages [15]. SDG Target 3.4 specifically seeks to reduce premature mortality from NCDs by one-third through prevention and treatment while promoting mental health and wellbeing [14, 15]. In 2021, NCDs accounted for over 43 million deaths globally, representing three quarters of all non-pandemic-related deaths, with 18 million of these deaths occurring among individuals under 70 years of age, exceeding deaths caused by injuries, infections (including COVID-19), and maternal and nutritional conditions combined. Notably, 82% of these premature deaths occurred in low and middle-income countries, with cardiovascular diseases accounting for over 19 million deaths annually, followed by cancers (10 million), chronic respiratory diseases (approximately 4 million), and diabetes (1.6 million).

In the same year, more than 700,000 people died by suicide, with many more attempting it [15]. Suicide remains a global issue, ranking as the third leading cause of death among individuals aged 15–29 years in 2021. It is not confined to high-income countries, as approximately 73% of suicides occurred in low- and middle-income countries that year. The WHO continues to focus on health-specific SDG targets, including ending preventable deaths, achieving universal access to reproductive healthcare, and combating infectious diseases. The organization provides technical assistance and capacity-building support to strengthen health systems and improve health outcomes globally.

### WHO Alliance for Transformative Action on Climate and Health (ATACH)

The WHO Alliance for Transformative Action on Climate and Health (ATACH) recognizes the interconnection between climate and health, as temperature extremes are associated with increased cardiovascular risk. Between 2024 and 2028, ATACH will continue to promote, support, and advance actions at the country level to build climate-resilient and low-carbon sustainable health systems [16]. This includes addressing supply chains and integrating nutrition into the climate-health nexus while identifying health-promoting actions in sectors such as water, food systems, social protection, trade, air, and energy. Currently, 93 countries and areas have formally committed to building climate-resilient and/or low-carbon sustainable health systems, with ATACH collaborating with over 80 non-state partner organizations. As part of the ATACH Strategy 2024–2028, Task Teams (TTs) have been introduced to focus on specific deliverables supporting country-level implementation of climate and health initiatives. On April 6th, 2025, the ATACH Task Team on integrating nutrition into climate-health policies was launched, aiming to develop guidance for enhancing the integration of nutrition into member states’ climate-health policies, identifying key entry points for policy action to generate co-benefits across sectors.

### The Lancet Commission. Sustainable Development Goals (SDGs) and Health Promotion

The Lancet Commission on Sustainable Development Goals (SDGs) and Health Promotion has established multiple commissions to address health-related SDGs, such as SDG 3 (Good Health and Well-being), while also tackling broader health issues aligned with the SDGs [4–6]. For example, the Lancet Commission on Pollution and Health assesses the health and economic impacts of pollution, while the Lancet Commission on Global Mental Health expands the global mental health agenda within the broader sustainable development framework. The Lancet Global Health Commission on High-Quality Health Systems in the SDG Era, comprising 30 academics, health system experts, and policymakers from 18 countries, emphasized the need for a "revolution" in health systems to achieve the SDGs [4]. The Commission advocates for health systems that consistently deliver high-quality care, maintain and improve health, build trust, and adapt to changing population needs, emphasizing values of efficiency, equity, resilience, and a focus on people.

1. Poor quality care is common across conditions and countries, with the most vulnerable populations receiving the worst care. The authors recommend universal health coverage (UHC) as a starting point for improving health systems’ quality alongside expanding coverage, especially for the poor, and financial protection.
2. High-quality health systems could save eight million lives annually in LMICs. The authors explain that 60% of deaths from treatable conditions are the result of poor-quality care.
3. Health systems should measure and report on the processes and outcomes that matter to people, including competent care, health outcomes, user experience and confidence in the systems. The authors call for “fewer, but better” measures of health system quality at national and sub-national levels, such as a dashboard of key health system performance metrics.
4. New research is essential to transform from low-quality to high-quality health systems. As an example, the authors propose research to evaluate the effects and costs of recommended improvement approaches on health, patient experience and financial protection.
5. System-wide action, including structural reform, is necessary to improve quality of care. The Commission recommends specific actions to improve quality of care across the health system.

The Commission identifies several opportunities for national governments, civil society, global partners and researchers to contribute to achieving high-quality health systems. For national governments, the Commission recommends: investing in health systems and making them accountable to people for their performance; partnering with other sectors, including education, transport and communication sectors, to create conditions for health system reform; embedding equality of care in UHC alongside; and improving measurement, among other actions such as Eat Lancet diet [18]. The Lancet Commission also quantifies the healthy diets for all, targets for sustainable food systems for all, and aims to mark scientific boundaries for decreasing degradation of environment caused due to food production [18]. The targets for the safe operating space of food systems including food production is among the largest drivers of global environmental alterations by contributing to climate change, loss of biodiversity, use of freshwater, interference with the global nitrogen and phosphorus cycles, and change in Earth system; such as chemical pollution, which is not assessed in this Commission.

### The protection of planet earth from environmental degradation and pollution

The Indian Vedic literature, considered to be the foundational text provides glimpses of environmental protection, ecological balance, weather cycles, hydrologic cycles, rainfall phenomena and related subjects through hymns and rituals. माता भूमि पुत्रोहं पृथिव्या”: Mata Bhumih Putroham Prithivyah, (~Atharva Veda,Bhumi Suktam 12-1-12, 5000 BCE) English Translation: Earth is my mother and I am her son.

Environmental degradation means the deterioration of the natural environment of the Earth, including the depletion of resources such as air, water, soil and the destruction of ecosystems, leading to reduced quality of life and threats to biodiversity, primarily driven by human activities like deforestation, pollution, overconsumption, and industrialization.Protection of the planet earth, is one of the most important SDGs, because earth provides soil and water as well grows plants, trees and crops, that are important for the survival of humans and animals. If there is degradation of the Earth, it may cause marked alterations in climate, soil and fertility of the land and decline in the growth of the crops and agri-foods. Planetary health is acutely under threat in the Anthropocene, with the causes and impacts of this threat inequitably distributed [4, 21]. Roughly 9 million premature deaths annually are linked to exposure to air and water pollution, 3·2 billion people are affected by land degradation, and many millions are affected by zoonotic disease, rising temperatures, and extreme weather events [22, 23]. People living in historically marginalised locations, especially people living in poverty, are particularly at risk. Economic growth trajectories (which dominate global economic policy) pose even greater risks through destabilisation of the global commons; biosphere, climate, and cryosphere, and nutrient and water cycles [21–24]. Integration of the concerns of socioeconomic into Earth-system boundaries (ESBs), may provide limits that should be adhered to in order to maintain the stability of the planet and health of the humans [25–28]. It may facilitate reaching a stable state of the Earth system and thereby promote sustainability for human health and wellbeing [27, 28].

### Food and Agriculture Organization (FAO). Sustainability of food systems and the planet earth?

Interestingly, three publications in Nature and Science have emphasized on safety of operating space [23] and earth system boundaries [24] with reference to providing guidance on human development because food and living space are crucial for existence of life and in turn that of earth. Food systems include the entire range of agencies, experts, farmers and consumers. The activities involved are in the production, aggregation, processing, distribution, consumption and disposal of food products that originate from agriculture, forestry or fisheries, and parts of the broader economic, societal and natural environments in which they are embedded. There are also systems such as farming, waste management system, input supply system, that interact with other key systems; energy system, trade system, health system. A sustainable food system (SFS*)* is a food system that delivers food security and nutrition for all in such a way that the economic, social and environmental bases to generate food security and nutrition for future generations are not compromised. This means that on the environmental dimension, sustainability is determined by ensuring that the impacts of food system activities on the surrounding natural environment are neutral or positive, taking into consideration water, soil, biodiversity, animal and plant health, the carbon footprint, the water footprint, food loss and waste.

The balance between the amount of foods coming from animals (animal-source foods) and from plants (plant-source foods) is an important component of healthy diets. Animal-source foods are a good source of highly bioavailable protein and key vitamins and minerals, but consumption of certain types and at certain levels has been linked to increased risk of diet-related NCDs. Consumption of plant-source foods has been linked to decreased risk of diet-related NCDs but diets consisting almost exclusively of plant-source foods may increase risk of nutrient deficiencies. A growing body of evidence also suggests that the food systems used to produce animal- and plant-source foods can impact the environment in different and significant ways, including the emission of greenhouse gases and water and land use. Additionally, unsafe food containing harmful bacteria, viruses, parasites or chemical substances causes significant illness and death globally, and both animal- and plant-source foods can be major sources of these contaminants.

### Sustainable diets and adverse impacts of animal sources of foods

WHO supports various countries in their efforts to promote healthier, safer and more sustainable diets as a priority through a variety of actions. Release of several guidelines on consumption of macronutrient and policy actions, activities to promote healthy diets in schools, sport events, and provision of scientific advice to the Codex Alimentarius through joint FAO/WHO programs such as the Joint Expert Committee on Food Additives (JECFA) and Joint Expert Meetings on Microbiological Risk Assessment (JEMRA). Building off this work, and to provide global guidance on optimal intakes of animal-source foods, WHO has brought together experts from all over the world, with a wide range of relevant expertise to develop various food systems.The animal sources of foods are highly bioavailable in nutrients, but commonly lacking globally, although can be important for food and nutrition security [8, 30]. Many populations in Sub-Saharan Africa and South Asia are getting benefit from increased intake of animal foods, due to improvement in nutrient intakes and decline in undernutrition. If such foods are consumed in greater amount, processed meat and red meat should be avoided, and saturated fat should be reduced to decrease the risk of NCDs [8–11]. This approach could also have several other benefits for sustainability of environment and the earth [5–7].

Production of animal foods may be associated with a large environmental impact [5–7, 29]. However, if food is produced at the appropriate scale and in accordance with local ecosystems and contexts, animal foods may have a role in circular and diverse agroecosystems [30–33]. Certain crops, in certain circumstances, can help restore the biodiversity and degraded land and mitigate greenhouse gas emissions from food production [29]. The amount and type of animal foods that is healthy and environmentally sustainable such as fish or seafoods, may depend on the local context and health priorities of the community and countries. The utility and necessity of such foods may change over time as populations develop, nutritional concerns evolve, and alternative foods from new technologies are easily available and acceptable [33]. The efforts of the agencies concerned with health and governments as well as civil society organizations, need to increase or decrease the consumption of animal foods which should be considered in light of the nutritional and environmental needs. In the local context and integrally, there is a need to involve the local stakeholders impacted by any changes. Policies, programs, and incentives are urgently needed to ensure best practices in production, curb excess consumption where high, and sustainably increase consumption where it is minimal.

Attempts to protect the Planet within the mindset of feeding an ever-growing human population, a sustainable plant diet are rather counterintuitive in regards to evolutionary evidence in support of the wild-type regimen (dietary omega6/omega-3 ratio 1:1) of omnivores, that has led us that far [33]. Animal-derived food correlates with much higher risk of development of NCDs, compared with plant-based food [11–13]. This was strongly confirmed from the results of 30 years of follow-up of 105,015 participants [11]. In addition to the negative effects on human health, healthy life-span and total longevity, the production and consumption of animal-source foods is increasingly associated with negative impacts on environment, climate, and health, that makes it unsustainable. Production of 1 kg of beef can cause 34.1–60.4 kg CO_2_-equivalent per kg of meat, still exceed the emission foot- prints of many plant-based foods such as wheat or rye bread (1.3 CO_2_-eq./kg), peas (0.8 CO_2_-eq./kg) or tofu (2.6 CO_2_ -eq./kg) [12, 13]. A Lancet Commission has proposed to quantify safe and just Earth-system boundaries (ESBs) and assess minimum access to natural resources required for human dignity and to enable escape from poverty [2, 21]. There is a description of a safe and just corridor on the earth planet, that may be essential to ensure sustainable and resilient health of human being, pets, and health of the planet earth and thriving in the Anthropocene [2, 23–25]. The safe and just corridor for planetary boundaries is a framework defining the necessary, living space for humanity, bounded by biophysical stability (safe) and minimal harm/equity (just). It indicates that 4 of 9 boundaries (climate, biosphere, nutrients, water) are crossed, requiring rapid transformation to limit the global warming.

### Plant based diets and traditional foods for health promotion and disease prevention

Lancet commission has described the healthy plant based diet which consisting of fruits, vegetables, legumes, whole grains, nuts, and unsaturated vegetable oils, including, a low to moderate amount of seafood and poultry, and including no or a low quantity of red meat, processed meat, added sugar, refined grains, and starchy vegetables such as potato and sweet potato [18]. Functional foods may be defined as foods that have certain components, which can address some markers in our body responsible for health or diseases [8]. Fruits, vegetable, nuts, whole grains, fortified foods, and foods containing probiotics or foods rich in omega-3 fatty acids and other nutrients are a variety of functional foods, which are components of plant-based diets [31–33]. Functional foods offer health benefits beyond basic nutrition. They often focus on consuming certain foods rich in bioactive compounds which may have a positive influence on specific body functions and potentially decrease the risk of diseases [8]. Functional diets may be defined as dietary patterns that prioritize the inclusion of functional foods to achieve specific health goals. Plant based diets are eating patterns including certain plant based functional foods to promote overall health and well-being. The approach of Food-as-medicine implies using food as a part of an individual’s health plan to prevent or help treat acute and chronic health conditions and diseases [31–33]. There may be trends and hurdles in the such intervention pyramid. However, such interventions may alter the future demand for specific food groups, their transport in supply chains, and the technologies used to process them. For example, Fabien De Meester used Sim’s hypothesis to feed flax seed and tea leaves to mother hen to produce healthy meat rich in omega-3 fatty acids and tea flavonoids, which have been used for health promotion [33].

Dietary interventions can help to prevent and treat most of the NCDs; including CVDs, T2DM, obesity, neurodegenerative diseases, bone and joint diseases and cancers [8, 33]. Further research may offer more individualized treatments depending upon concerned nutrient deficiency. It may be challenging given the inter-individual variability and complexity of the body’s response to food and related factors, such as dietary habits, genetics, lifestyle, and biosphere. Quantification in health improvements may be essential to prove the added value of more individualized medicinal food interventions compared with adopting a general healthy diet. This approach may cause a shift to produce more health-promoting foods, including whole foods, minimally processed foods, and selected processed foods. The food processing industry and supply chains must adapt to these new scenarios, which would be challenging to food industry. Auxiliary technologies and methods are enablers, including delivery services, wearable technology, health-monitoring apps, and data-driven consumer behaviour analysis.

Before going into the details of 12 basic characteristics, it is important to remember the three basic interlinked physiological needs of the healthy food: (i) Variety – to help achieve a nutritionally adequate diet and help protect the biodiversity of food systems. (ii) Balance – to help reduce risk of diet-related non-communicable diseases and excessive use of finite environmental resources and production of greenhouse gas emissions. (iii) Moderation – to help achieve a healthy body weight and avoid wasting finite environmental resources used in providing food surplus to nutritional requirements [19]. These prerequisites, have also been described below.

### The twelve basic characteristics of a healthful diet

High-quality Indo-Mediterranean type of diets are also mainly plant based diets that are characterised with unrefined, unprocessed functional foods, whole grains such as dry millets, grams, peas, beans and porridge [34–37]. Vegetables such as leaves of fenugreeks, spinach, coriander, radish, mustard and gourds, nuts such as almonds, walnuts, peanuts, pistachio, and fruits such as apples, grapes, water melon, papaya, guava, musk melon, etc. are major components of this diet [35]. It seems that healthy vegetable oils such as olive, mustard, sun flower, rape seed oil and protein sources that are beneficial; millets, Bengal gram, beans, pulses, cottage cheese, are crucial in this diet. Among animal sources, white meats; such as sea foods and dairy products should also be part of this diet [35]. Vegetables and spices with medicinal properties; gourds, turmeric, fenugreek, coriander, cumin, cloves, cinnamon, and cardamom may also be called high quality nutraceutical foods, respectively [34]. There is a need to find out traditional foods from all other countries which may be functional, protective and healthful [36]. Most of the traditional foods possess many of the given characteristics of the diet such as slow absorption, diversity in foods, high nutrient density, high fibre, moderate fat and low in salt, sugar and trans fat. Table 2.

**Table 2:** The twelve qualities of the high quality healthy diet or plant based diet

Qualities of foods	Examples of foods
1. Slowly absorbed foods	Nuts, vegetables, whole grains
2. Food diversity	Whole grains, beans, vegetables, fruits
3. High nutrient density	Nuts, vegetables, whole grains
4. No trans fat	Grilled foods, boiled foods
5. No/low sugar refined	Use dates, apples, papaya, oranges, raisins
6. Low salt	Fruits, vegetables, nuts
7. Moderate fat	Nuts, pulses, beans, green leaves
8. High fibre	Vegetables, whole grains, fruits
9. Beneficial effects on gut microbiota	Vegetables, whole grains, fruits
10. Non per-oxidised foods	Fresh foods, without frying
11. Spices, 15–30 g/day	Turmeric, cumin, coriander, fenugreek, cinnamon, cardamom, cloves
12. Foods requiring mastication & helpful in hydrogen production	Whole grains like porridge, nuts, fruits, vegetables, and millets

Functional foods are those foods that are rich sources of micronutrients such as antioxidants, vitamins, minerals, essential and non-essential amino acids and omega-3 fatty acids which allow these foods to possess some physiological function on their consumption [36–41]. Recently, Menichetti et al., presented the chemical complexity of functional foods and implications for therapeutics, emphasizing that, certaine foods have natural dark matter (NDM), which can modulate the genes and proteins, providing good health. Whereas, poor nutrition has broad societal effects, driving up health care costs and decreasing productivity [36, 37], adopting a healthy plant based diet and healthy lifestyle can significantly counteract even a strong genetic predisposition to CAD and reduce the relative risk nearly by 50% [36].

### Comparison of various types of diets, low in meat and high in plant foods

It seems that majority of the healthful diets are plant based diets with moderate amount of meat and dairy products, which serve the requirement of proteins and nutrients. There is evidence from epidemiological, long-term prospective observational studies, and short-term trials of intermediate outcomes, supporting evidence that western-type diets have adverse effects, whereas Mediterranean type of diets rich in nutrient rich foods, with a modest amount of fish are protective against CVDs, T2DM, and cancer [33–38]. It is well known, that high-quality diets are characterized by unrefined, unprocessed, or minimally processed foods with a low or moderate glycemic index and are plant based [37]. It is food diversity that determines the differences in variety of food items such as vegetables, fruits, nuts, whole grains, spices, fish, and vegetable oils, which are beneficial and dense in nutrients. However, western types of foods, consisting of refined and fast foods, syrups, red meat, processed meat, salt, sugar and trans fat, have been found to predispose potential causal relationships between specific dietary component and CAD, T2DM and cancers [37–39]. Singh et al. have compared the Indo-Mediterranean, Japanese and Mediterranean diets, with the DASH diet, that are known to cause health promotion and disease prevention [37]. The people in countries eating Mediterranean type of diets, have a low risk of NCDs [33–38]. This observation is identical with Japanese populations, who have longest life expectancy, as well as a healthy whole life course [37]. The Mediterranean diet is characterized by greater consumption of whole grains, beans, whole wheat bread, vegetable and fruits, along with olive oil and fish, but low red meat [37–39]. The DASH diet proposed for lowering blood pressure, shares potassium rich fruits and vegetables, with the Mediterranean type of diet because it is also rich in fruits, vegetables, nuts, seeds, whole grains, and fish, with lower content of red and preserved meat and poultry [37]. The Japanese diet also includes increased content of whole rice (whole grains), fish, vegetables, sea-weeds and fruit, but with lower intake of fats and oils [37]. It seems that all these four types of diets are mainly plant based but, Mediterranean, DASH diet and Japanese diet contain moderate amount of animal foods. Therefore, if the animal foods, in particular red and processed meats are excluded from these diets, they may be modified as plant based diets. There is a need to determine the effects of changes in the content of animal foods and western-type processed foods in these diets, on the risk of NCDs [41]. The differences in these diets are given in Table 3.

**Table 3:** Comparison of Indo-Mediterranean diet with other scientific diets

Foods	Indo-Mediterranean Diet	Mediterranean Diet	DASH Diet	Japanese Diet
Vegetables, fruits	400 g/day	High	High	High
Nuts	50 − 100 g/day	High	Moderate	Low
Whole grains, beans	400 g/day, high; beans millets, porridge, grams, soybean, green beans	Moderate, legumes	Moderate, legumes	High rice, soya bean, tofu
Vegetable oil	30 − 80 g/day, mustard oil or blend of olive oil	Olive oil, high (100 g/day)	Low saturated fat foods, oils	Low rice bran oil
Fish	100 − 150 g, twice/week	Moderate	Moderate	High, raw
Dairy products	Buttermilk and curd	Low fat	Low fat	Low
Wine	Not advised but allowed	Moderate	Not advised	Sake, rice wine
Spices (coriander, cumin, turmeric, cloves, cardamom)	High (50 − 150 g/day), coriander, cumin, turmeric, fenugreek	Not advised	Not advised	Not advised
Poultry	Not advised	Moderate	Low	Low
Red meat	Not advised	Low	Low	Low
Preserved meat	Not advised	Low	Not advised	Low
Sweets and sugar	Not advised	Low	Low	Low
Nutrients	High flavonoids, fiber, K, Mg, Ca, iron, proteins	No specific advice for protein	High K, Mg, Ca, fiber, protein	High *n*−3, K, Mg, Ca, high protein
Food diversity	Marked	Moderate	Moderate	Moderate
Glycemic index	Very low	Lower	Lower	Very low

These diets differ in many aspects as given in the table. The Indo-Mediterranean-style diet is characterized with excess of whole grains, millets, grams, peas, porridge, green beans, and a variety of spices; fenugreek, cumin, turmeric, coriander, cardamom, cinnamon, black pepper, saffron and cloves, indicating marked diversity and low glycemic index of the diet. This diet can modulate health and prevent diseases as well as health of the planet because there is hardly any type of meat in this diet..(Modified from Singh et al., 2022, reference 37)

K = potassium, Mg = magnesium, Ca = calcium, *n*−3 = long-chain omega-3 fatty acids

### Plant based diet and risk of non-communicable diseases

Majority of the above healthy diets have a pattern of foods that are plant based, which contributes to the quality of plant based diets. In a meta-analysis of 14 studies involving 976,396 participants [41], the findings revealed that a plant-based diet (PDI) can decrease cancer mortality (RR = 0.88, τ^2^: 0.02, I^2^: 84.71%), CVD) mortality (RR = 0.81, τ^2^: 0.00, I^2^: 49.25%) and mortality (RR = 0.84, τ^2^: 0.01, I^2^: 81.99%) risk. Interestingly, increased consumption of healthy plant-based diet was negatively correlated with cancer mortality (RR = 0.91, τ^2^:0.01, I^2^:85.61%), CVD mortality (RR = 0.85, τ^2^: 0.02, I^2^: 85.13%) and mortality (RR = 0.85, τ^2^: 0.01, I^2^: 89.83%). However, an unhealthy plant-based diet was positively correlated with CVD mortality (RR = 1.19, τ^2^: 0.02, I^2^: 80.03%) and mortality (RR = 1.18, τ^2^: 0.01, I^2^: 89.97%) and correlated with cancer mortality (RR = 1.10, τ^2^: 0.03, I^2^: 93.11%) without contradictory results. It is clear that the healthy plant based diet may be negatively associated with all-cause mortality, whereas the unhealthy diet was positively associated with all-cause mortality. In a systemic review, vegetarian and vegan diets were significantly associated with glycemic control, better lipid profile, body mass index, body weight, inflammation, and lower risk of CAD and cancer [42]. Vegetarian diet was also associated with lower deaths from CVDs. However, in the pregnant women, no difference in the risk of developing gestational diabetes and hypertension were reported following vegetarian diets. It is possible, that plant-based diets may be beneficial in decreasing risk factors, as well as risk of CVDs, cancer and mortality [42]. There is a need to have, caution before broadly suggesting the adoption of these diets, since the strength-of-evidence of study results is significantly limited by the large study heterogeneity alongside the potential risks associated with potentially restrictive regimens.

It is difficult to suggest mitigation of risk strategies, because pro-atherogenic lipid, lipoprotein, and inflammatory processes may facilitate the development of atherosclerosis. Therefore, an understanding of the relation between plant based diets and these mechanisms may be crucial [43]. In a meta-analysis, 43 studies on plant based diets and risk of risk factors were included. Plant-based diets were generally associated with favorable lipid and lipoprotein profiles, showing reduction in total cholesterol, low-density lipoprotein cholesterol, and apolipoprotein B levels, with a decreased low-grade inflammation, and reduced C-reactive protein levels. Plant based diet interventions showed greater effects compared to habitual dietary patterns, and for non-low-fat vegan and controlled dietary therapies. The associations between plant food groups and outcomes of CVD and of certain biomarkers, and the potential mechanisms underlying associations between diets and reduced CVD risk were clear.

### Mechanisms of beneficial effects of plant based diets

The beneficial effects of the prudent dietary pattern may stem from the plant based diets which are rich in nutrients with high content of alpha-linolenic acid (ALA), antioxidants such as polyphenolics and flavonoids and carotenoids, vitamins, and minerals, essential and non-essential amino acids present in the diet [34, 38, 39, 44]. Omega-3 fatty acids, such as ALA is rich in mustard oil, walnuts, sea weeds, green leaves, whole grains, and seeds; eicosapentaenoic acid (EPA) and docosahexaenoic acid (DHA) are rich in fish and fish oil and can be beneficial in the microcirculation and cardiac rhythm, with implications for myocardial infarction [99, 100]. These nutrients are known to decrease inflammation, insulin resistance and blood lipids in several studies [34–39]. Esposito et al. [45] randomized 180 patients (99 men, 81 women) with metabolic syndrome to a Mediterranean-style diet, characterized by whole grains, vegetables, fruits, nuts and olive oil vs. a cardiac-prudent diet with fat intake less than 30%. After a follow-up of 2 years, subjects in the intervention diet showed a greater weight loss, lower C-reactive protein and pro-inflammatory cytokine levels, less insulin resistance, as well as lower total cholesterol and triglycerides and higher HDL cholesterol. The prevalence of metabolic syndrome was reduced to one half. Since inflammation, hyperlipidemia, hyperglycemia, free-radical stress and insulin resistance are basic mechanisms responsible for CVDs and other chronic diseases, a plant based diet could protect against these problems. A recent study showed that exogenous plant MIR168a specifically targets mammalian LDLRAP1, which is evidence for cross-kingdom regulation by microRNA in humans, providing further evidence regarding the mechanism of diet in the prevention of NCDs [46].

**Fig. 3:**
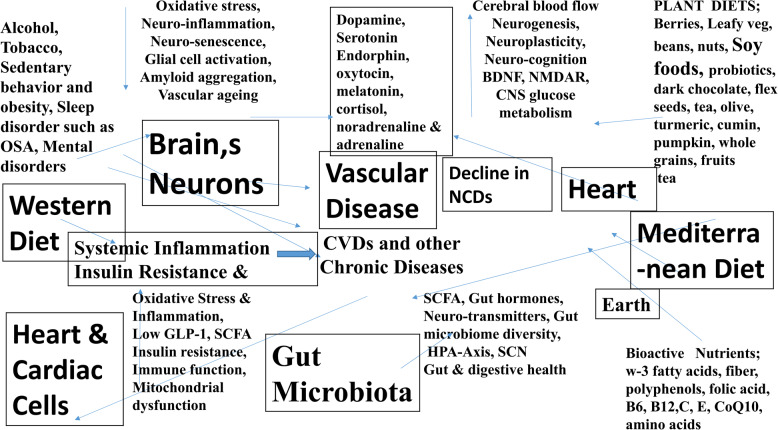
Mechanisms of adverse effects of western type of diets with animal source of meat and of benefits due to intake of plant based sustainable diets leading to decrease in non-communicable diseases (NCDs), and improved health of the planet Earth. CVD = cardiovascular diseases

In a meta-analysis including 141 studies, [47], the intake of protein was lower in people eating plant-based diets compared to meat-eaters, although it was within recommended limits. The intake of fibre, polyunsaturated fatty acids (PUFA), folate, vitamin C, E and magnesium was greater, eicosapentaenoic acid (EPA) and docosahexaenoic acid (DHA) intake was lower in vegetarians and vegans as compared to meat-eaters. The consumption of vitamin B12, vitamin D, iron, zinc, iodine, calcium and bone turnover markers were generally lower in plant-based group, compared with meat-eaters. Vegans had the lowest vitamin B12, calcium and iodine intake, and also lower iodine status and lower bone mineral density. People eating meats were at risk of inadequate intakes of fiber, PUFA, α-linolenic acid (ALA), folate, vitamin D, E, calcium and magnesium. The nutrient inadequacies were found across all dietary patterns, including vegetarian, vegans and meat-based diets. It is apparent that, despite nutrient inadequacy, plant-based diets are generally better for health and the environment [47]. Therefore, the public health strategies should facilitate the transition to a balanced diet with more diverse nutrient-dense plant foods through consumer education, food fortification and by using food supplements [45, 48]. Adverse effects of western type of diets and lifestyle, and benefits of plant based diet on concerned systems, indicating mechanisms are given in the Fig. 3.

### Development of functional foods by the industry for sustainable plant based diets

It seems that development of a functional food by the food industry should aim to add certain nutrient rich foods or nutrients in the food, which can take care of the nutrient inadequacy of the sustainable plant based diets. These foods should possess all of the characteristics of a healthy plant based diet such as proteins and micronutrients to provide physiological function for health promotion and disease prevention. There are alternatives to replace conventional animal foods; meat, dairy, and seafood with novel plant-based alternatives (PBAs). It seems that consuming more sustainable diets, in contrary to western food systems, plant based alternatives are processed foods that aim to mimic the structure, texture, and sensorial properties of the animal foods, which are intended to replace. It is possible that they can support transitions towards more plant-based diets among consumers who strive to decrease the animal food intake but are not willing to compromise convenience or desirable sensory attributes. Therefore, plant based alternatives are often based on western food system such as legumes and nuts they differ in the way, they are processed and in their sensory profiles. The development of processed foods may provide added functional benefits to consumer’s well-being [47]. Intrinsic regulation and end-of-use purposes in different countries, worldwide meanings and definitions of this term are still not clear. Hence, there is a need to standardize this definition and propose a guideline to attest that some ingredients or foods truly deserve this special designation.

The focus should be directed at the practical guidelines that can be used to develop and test the efficacy of ingredients in the potential functional foods. The most widespread functional ingredients, such as flavonoids, vitamins and other antioxidants, polyunsaturated fatty acids (PUFAs), probiotics or prebiotics or synbiotics, proteins and their technological means of delivery in food products are crucial to maintain plant based status of sustainable diets. There is an urgent need for large-scale dietary changes in the western type of diets to achieve sustainable and resilient food systems within the planetary boundaries while ensuring nutritious diets for a growing world population [48]. Interestingly, modelling studies have reported that the shift to more plant-based diets would mitigate the environmental pressures from food systems while promoting public health. These studies emphasize to substitute animal-source foods by whole foods such as nuts, seeds, raisins, strawberries, millets, soya products and legumes, which are known to provide taste and flavor that are key barriers to move away from animal foods. Substitution of whole foods maintains the taste, as well as convenience, and cultural values of eating [48]. This is important because eating vegetable proteins compared to animal source of protein may be associated with significant decline in mortality [49].

### Soya bean as major substitute for animal source of foods, in the plant based diet

In view of the rapidly growing global population and increasing demand for protein, soy protein can replace animal protein in the future because it is sustainably produced and has a balanced composition of essential amino acids [50–54]. In addition, millets, seeds, nuts and spices are other functional foods which may be added to provide texture, taste and flavour in the final products. It seems that, pure soy protein hydrogels have several problems, such as insufficient mechanical properties, which limit the application of soy protein hydrogels in the food field [50, 51]. An effective method of protein compositing may improve the gel properties of soy protein, that depends on the specific needs, for a suitable gel preparation to realize the wide application of composite soy protein gel systems in the food industry. In addition, high technology should be used to maximize the value of protein while paying attention to the odour and allergenicity in soy protein. The limitations of the current composites with other proteins such as millets and beans should be realised to minimize the drawbacks of soy protein and maximize the utilization of protein resources to meet the demand for protein. Sulaeman and colleagues have conducted several studies to explore the health benefits of soybeans and soybean products. The particular focus was on the role of soya bean protein in preventing and treating NCDs. The research often highlights the potential of soy-based foods; tofu, soya bean bolls and soya bean flour to make bread, tempeh and soymilk to positively impact various aspects of human health [50–54].

The techniques of germination and fermentation of tempe were used to improve the concentrations of these bioactive metabolites [50]. There was a significant improvements in the content of Amino acid, however, germination process showed only mild improvement in bioactive metabolites. Interestingly, tempe fermentation showed significant increase in the concentrations of genistein, daidzein, glycitein, acetyl genistin, acetyl daidzin, 3-hydroxyanthranillic acid, and meglutol (> twofold increase with p < 0.05). In addition, there was significant improvement in the amino acid levels. Soybean products, such as soya bean balls, tofu, tempeh, edamine and soy milk are well known high quality protein products which have become popular for consumption in Asia [51]. Apart from these products, soya bean flour in combination with barley and millets (one third each) is also used to make Indian bread, to provide proteins and fatty acids. Soya beans are also a good source of calcium, iron, and zinc as well as essential and nonessential amino acids and fatty acids. Compared to other plant-based proteins such as whey, milk pea and barley, soy protein, is considered to have a high protein quality [54]. Soy and pea are legumes without sulfur-containing amino acids such as methionine and cysteine, and like barley that have a limited amount of lysine. Among the three plant-based proteins, soy protein is the one that has similar amino acids composition compared to milk and whey protein but whey proteins contain a lesser amount of histidine.

Soya foods are recognized for their potential health benefits, including reducing the risk of CAD, diabetes and certain cancers, e.g. breast and prostate, and improving bone health [50–54]. In addition, soy alleviates hot flashes and may favourably affect renal function, alleviate depressive symptoms and improve skin health. The beneficial effects of soy foods appear to be due to isoflavones, which are classified as both phytoestrogens and selective estrogen receptor modulators. The concerns about adverse effects are based primarily on animal studies, whereas the human research supports the safety and benefits of soy foods. The recent conclusion of the European Food Safety Authority confirms that isoflavones do not have an adverse effect on the breast, thyroid or uterus of women. However, there may be potential interactions with certain medications and possible effects on hormone levels, particularly in breast cancer survivors.

### Traditional soy bean food intake in Asia

Records in history indicate that the soya bean was used as a food originated in China possibly around 2,000 years ago [55]. However, archeological evidence suggests that soybean domestication may have occurred several thousand years earlier [56]. Soybean as food spread from China to Japan and other Southeast Asian countries like India, Indonesia, although recent data indicate that there might have been multiple independent efforts to domesticate wild soybeans in these countries [57]. There are two general categories of Asian soya bean foods, fermented and unfermented. Fermented soy foods include natto, tempeh and miso, whereas unfermented foods include soymilk and tofu. Soya bean flour along with millets and barley is being used in India to increase protein intake among vegetarians (Personal communication, RB Singh). Tempeh is a more recent creation of Indonesia which has been developed in around the year 1600 s [58]. Most of the soya bean consumed in the world, is in the unfermented form, excluding soy sauce, which is a condiment and the intake is still least in the western world [54]. Recently, education has been found to be most important determinant of lower mortality [59]. However, in the western world, people are educated but are not health educated and motivated [60], resulting in to only modest modification in the diet and lifestyle. In addition, despite education, there is little change in the intake of plant based diets and use of soya bean products in the major meals in most of the advanced countries [61]. Plant based diet may also influence mitochondrial dysfunction [62]. Since, poverty is not the absolute cause of deaths due to NCDs [63], building a healthy urban design index (HUDI) may be suggested to promote health and sustainability in European cities [64] which may be ideal for the whole world.

**Fig. 4:**
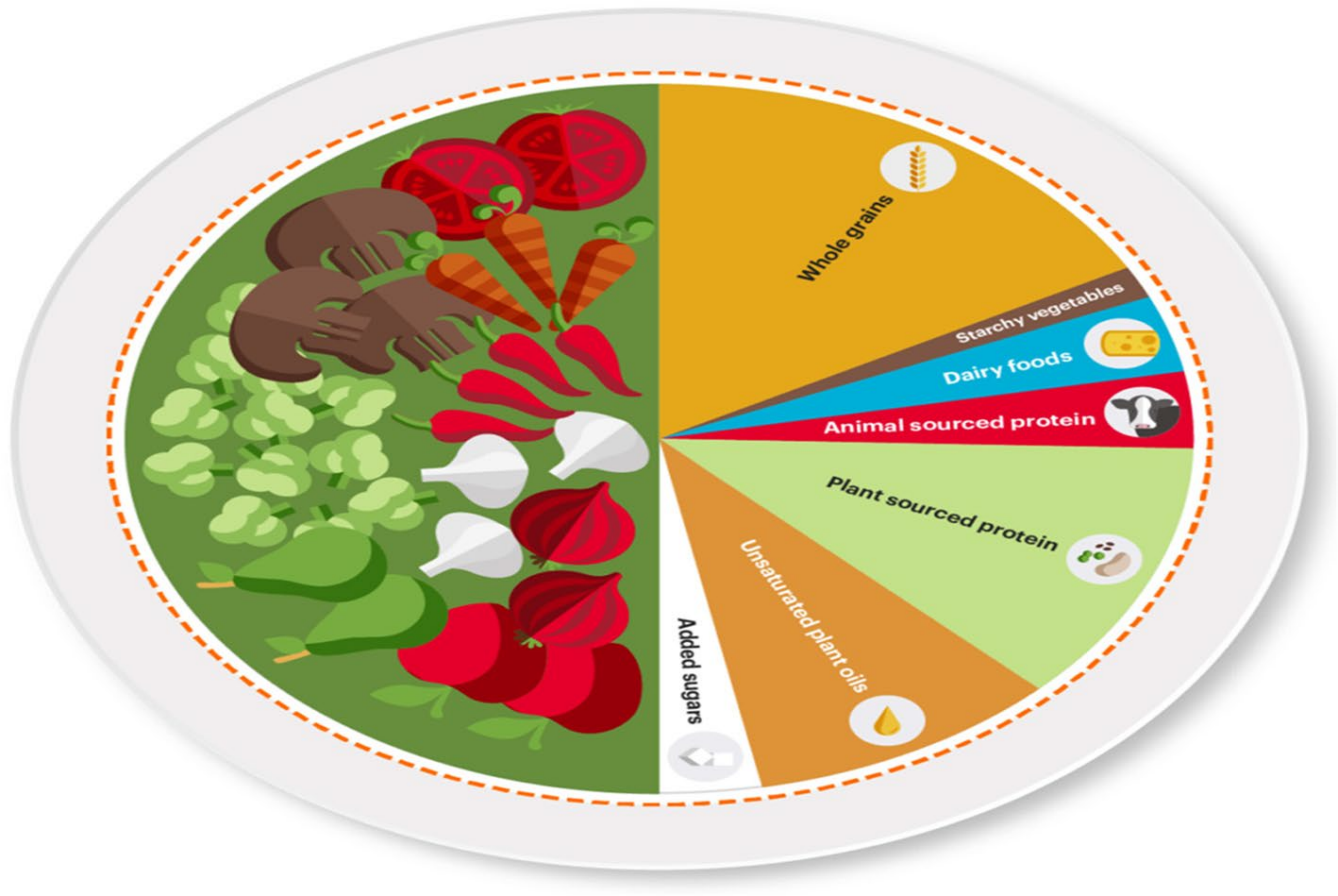
The Eat Lancet diet (Adapted from Willet et al., reference 18)

The EAT-Lancet diet or the Planetary Health Diet, includes the dietary guidelines that are designed to promote both sustainability of environment and human health [18, 65]. This diet is characterised with a shift towards plant-based functional foods while decreasing the intake of meat, in particular preserved and red meat [66, 67] and other food choices such as decline in the intake of sugar, saturated fat and trans fat that are known to have lower sustainability but with adverse effects on health. The plant based foods are shown in multiple colours. Figure 4.

### Affordability and feasibility of modified plant based diet

Recent reviews indicate that there is strong evidence through epidemiological, long term prospective follow up studies and randomized controlled trials that a modified plant based Mediterranean type of diet can decrease morbidity and mortality [9, 37, 41, 60], International College of Nutrition also advises traditional and seasonal vegetables, nuts and fruits which are more feasible and affordable to buy, by all middle and lower social classes to serve the needs of plant based diets [8, 18, 37, 61, 70]. Adding soya bean products such as tofu, tempe and bread, as protein substitutes, to modified plant based diet can further decrease the need for red meat intake. Finally, Indo-Mediterranean-style dietary pattern, including fruits, vegetables, seeds and nuts (400–500 g/day) and another 400–500 g whole grains including soya bean products (bread, tempe, tofu as), along with 30–50 g vegetable oils such as soya bean, mustard, canola or olive oil may be protective against NCDs [61, 70, 71]. There is an urgent need for a global transformation of the food system, which may lead to decreased biological ageing and increased life expectancy [65]. In the UK biobank cohort study, among 141,562 participants (56.02 ± 7.94 y; 45.12% male), adherence to the EAT-Lancet diet is linked to delayed biological ageing and increased life expectancy [66]. If high-income countries begin to consume plant-based diets, it would cut emissions of greenhouse gas as well as sequester almost 100 billion tons of CO2 by 2100 [66], in the case where the land was used to farm animals and reverted to its natural state. Apart from food and nutrient content of diet, time of eating and missing breakfast are chonobiological risk factors of CMDs. Franz Halberg established that the effects of diet on body weight can be enhanced if the meal is taken as breakfast [68, 69] or in a time window before sunset, because eating dinner in particular late at night can cause increased risk of CMDs. Thus, other interventions are also important to reduce the risk of NCDs [70]. Some experts have also emphasized the role of Eight Fold Paths of Buddha [72], educational attainment (1970–2030) to evaluate SDG 4 progress [73, 74] to be crucial to support the SDGs.

Green hydrogen, produced via renewable-powered electrolysis, offers a sustainable solution for agriculture, aiming to decarbonize fertilizer production and reduce reliance on fossil fuels. Recent innovations allow for, producing carbon–neutral hydrogen from farm waste water electrolysis, while, microbial, hydrogen-based food production presents a highly efficient high yield alternative to traditional farming [74]. These technologies, as highlighted in the recent International Congress on Hydrogen in Medicine and Biology, are crucial for, reducing emissions in hard-to-abate sectors and supporting, global sustainability goals, because use of H2 in irrigation water can enhance 20% increase in food production [75].

## Conclusions

In brief, SDGs of the UNO aim to decrease the intake of animal source of foods to protect the planet earth and environment as well as changes in climate. Therefore, plant based diets are advised to consumers, which are components of Mediterranean-style diet or Indo-Mediterranean-style dietary pattern, including fruits, vegetables, seeds and nuts (400–500 g/day) and another 400–500 g whole grains including soya products, along with 30–50 g vegetable oils such as soya bean, mustard, canola or olive oil. These dietary recommendations of this paper are closely related to the food system reform promoted by the Lancet Commission and implemented by WHO and UNO.

These foods may be substituted for western pro-atherogenic foods, such as red meat and processed meat, in conjunction with lower amount of healthy meat such as fish and other sea foods. Plant based diets with low meat, may be protective against CVDs and diabetes as well as other chronic diseases in most low- and high- middle-income and high-income populations of the world who are highly susceptible to NCDs. Plant based diets may cause decreased intake of proteins, iron, folate and vitamin B12. Therefore, there is a need to develop plant based protein substitutes, such as soya bean based food products; tofu, tempeh, edamine, soya bean bolls, soya bean milk and soya bean flour to make bread and biscuits to increase the intake and ensure optimal protein supplementation. Health policies should create conducive environments for making healthy choices affordable and available, as well as improving air quality along with other interventions that are essential for motivating people to adopt and sustain healthy behaviours to protect the planet and serve the SDGs of the UNO.

## Data Availability

Not applicable as this a Review Article.
